# Simultaneous Determination of Seven Phenolic Acids in Rat Plasma Using UHPLC-ESI-MS/MS after Oral Administration of *Echinacea purpurea* Extract

**DOI:** 10.3390/molecules22091494

**Published:** 2017-09-07

**Authors:** Yan Du, Zhibin Wang, Libo Wang, Mingjie Gao, Liqian Wang, Chunli Gan, Chunjuan Yang

**Affiliations:** 1 Department of Medicinal Chemistry and Natural Medicine Chemistry, College of Pharmacy, Harbin Medical University, No. 157 Baojian Road, Nangang District, Harbin 150081, China; duyan_0525@163.com (Y.D.); wanglibo66@sina.com (L.W.); 2 Key Laboratory of Chinese Materia Medica (Ministry of Education), Heilongjiang University of Chinese Medicine, Harbin 150040, China; wzbmailbox@126.com; 3 Department of Pharmaceutical Analysis and Analytical Chemistry, College of Pharmacy, Harbin Medical University, No. 157 Baojian Road, Nangang District, Harbin 150081, China; gaomingjie8888@163.com (M.G.); wangliqian93@163.com (L.W.)

**Keywords:** *Echinacea purpurea*, phenolic acids, UHPLC-ESI-MS/MS, pharmacokinetics

## Abstract

A rapid and sensitive Ultra High Performance Liquid Chromatography Electrospray Ionization Tandem Mass Spectrometry (UHPLC-ESI-MS/MS) method was developed and validated to simultaneously determine the concentration of seven phenolic acids (syringic acid, ferulic acid, caffeic acid, vanillic acid, *p*-coumaric acid, 3,4-dihydroxybenzoic acid and 4-hydroxybenzoic acid) in rat plasma after oral administration of *Echinacea purpurea* extract. After mixing with the internal standard (IS), butylparaben, plasma samples were prepared by liquid–liquid extraction with ethyl acetate. The separation was performed using the Agilent Eclipse Plus C_18_ column (1.8 μm, 2.1 mm × 50 mm) with a gradient system consisting of solution A (0.1% acetic acid in water) and solution B (methanol) at a flow rate of 0.3 mL/min. The detection was accomplished by a multiple reaction monitoring (MRM) mode with electrospray ionization (ESI). The method was validated in terms of linearity, precision, accuracy, extraction recovery, matrix effect and stability. This method was successfully applied to study the pharmacokinetic properties of the seven compounds after oral administration of *Echinacea purpurea* extract in rats.

## 1. Introduction

*Echinacea purpurea* (*E. purpurea*) is a perennial herb that belongs to the Asteraceae family, and it is native to North America. It has become one of the most popular herbal medicines in the international herbal market. The products that use *E. purpurea* as their main raw material have great potential use [1]. The history of its medicinal use can be traced back to the Indian period of North America, and it has been known for treating insect and snake bites, eczema and tuberculosis [2]. Nowadays, a number of researchers are focused on its pharmacological activities, such as immunomodulatory activity, as it is now shown to be effective in the prevention and treatment of the common cold, coughs, bronchitis, and respiratory infections [3,4]. In addition, it has also exhibited strong anti-viral, anti-bacterial, anti-fungal, anti-oxidant, anti-carcinogenic, anti-inflammatory and wound healing effects [5,6].

The immune response of *E. purpurea* is to combat pathogen infections and enhance the activities of neutrophils, macrophages, poly morphonuclear leukocytes (PMN) and natural killer cells (NK) [7]. Especially in some African countries, it could have a great effect on treating patients who are infected with HIV [8]. Clinical trials have demonstrated that *E. purpurea* can be used for the treatment of cancer or provide a substitute treatment for cancer patients [9]. In addition, *E. purpurea* extract can enhance the activity of CD34+ stem cells in mice with acute myocardial infarction (AMI), which may help in stem cell-based regeneration of the infarcted myocardium [10]. It was found that alkylamides in *E. purpurea* have anti-depressant effects [11,12,13,14].

**Figure 1:**
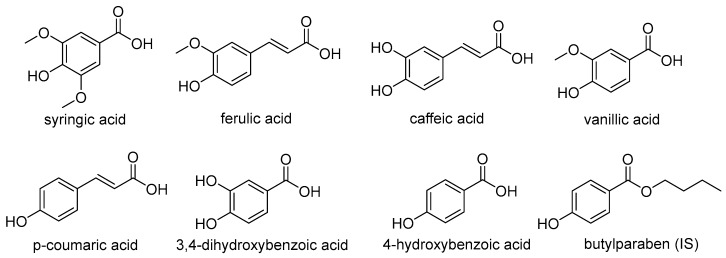
The chemical structures of the seven analytes and the internal standard (IS), butylparaben.

*E. purpurea* also contains a variety of chemical components such as phenolic acids, saponins, flavonoids, alkaloids, polysaccharides, glycoproteins and volatile oils [15,16]. Phenolic acids have been reported as the major effective components in *E. purpurea*. These include syringic acid, ferulic acid, caffeic acid, vanillic acid, *p*-coumaric acid, 3,4-dihydroxybenzoic acid, 4-hydroxybenzoic acid (Figure 1), cichoric acid, chlorogenic acid, quinic acid, and so on [17,18]. On the basis of previous studies, phenolic acids such as cichoric acid, chlorogenic acid and quinic acid have already been determined in rat plasma, so these compounds were not focused on in this work [18]. A series of research showed that they have powerful anti-oxidant and free radical scavenging ability, such as superoxide anion free radical (O_2_^•−^), hydrogen peroxide (H_2_O_2_), hydroxyl radical (•OH) [19,20]. The mechanism of the anti-oxidant effect illustrated that it can directly absorb free radicals, inhibit the activity of oxidase and enhance the activity of SOD [21]. It was demonstrated that phenolic acids have an inhibitory effect on α-amylase, α-glucosidase associated with type 2 diabetes and the angiotensin converting enzyme (ACE), which suggest that it may provide a new method for the treatment of hyperglycemia and hypertension in some ways [22]. In addition, phenolic acids can treat anti-obesity and upper respiratory tract infections (URTIs) [23].

Various analytical methods, including high performance liquid chromatography-ultraviolet (HPLC-UV) [24], high performance thin layer chromatography (HPTLC) [25], colorimetry [26], liquid chromatography-tandem mass spectrometry (LC–MS/MS) [9] and UHPLC–MS/MS [27] have been used in the qualitative or quantitative analysis of these phenolic acids. However, these methods have mainly focused on the general components with high concentrations in *E. purpurea*. This work developed a rapid and sensitive method for the simultaneous determination of syringic acid, ferulic acid, caffeic acid, vanillic acid, *p*-coumaric acid, 3,4-dihydroxybenzoic acid and 4-hydroxybenzoic acid in rat plasma, and it may help to evaluate the pharmacokinetic properties of *E. purpurea* effectively. Meanwhile, this is the first study to describe these phenolic acids using UHPLC-ESI-MS/MS in rat plasma after oral administration of the *E. purpurea* extract.

## 2. Results

### 2.1. UHPLC-MS/MS Optimization

Because of the hydrophilic properties of phenolic acids, a reverse phase system was used for the method development. Thus, the organic mobile phase combined with a buffer was selected to be used in this study. The response of the analytes had a higher signal using methanol–water than using acetonitrile–water, so the methanol–water was selected as the basis for the further mobile phase development. Formic acid, acetic acid and ammonium acetate were tested by adding them into the water mobile phase in order to improve the peak shape and enhance the sensitivity of the analytes in negative ionization mode. The results showed that 0.1% acetic acid in methanol–water exhibited the best peak shape and sensitivity. The further optimization of the ratio of the organic phase and buffer was also conducted. After continuous and appropriate adjustment of the chromatographic separation conditions, the best results were achieved by a gradient elution program including methanol–water (0.1% acetic acid) at a flow rate of 0.3 mL/min with the run time at 8 min.

**Figure 4:**
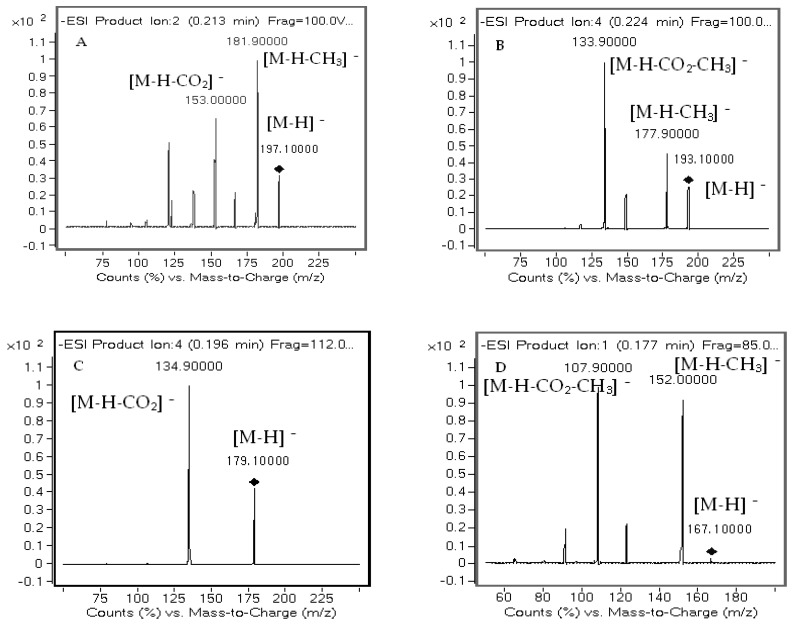

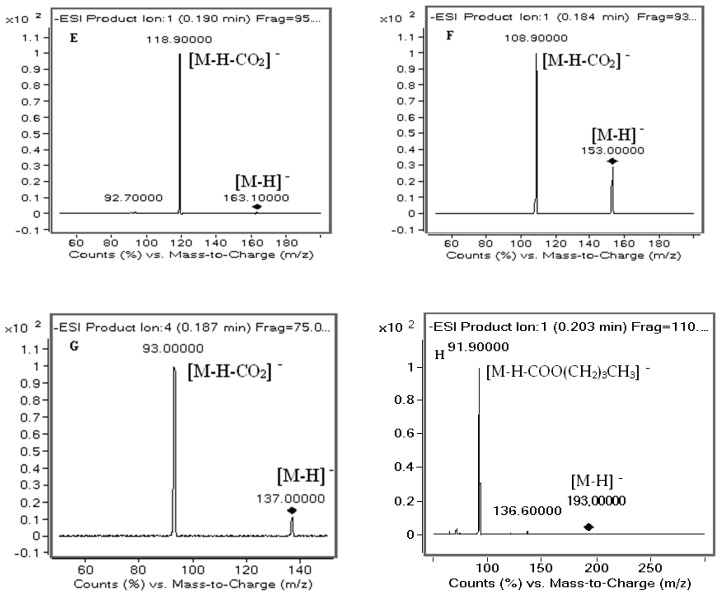
Product ion mass spectra of the seven analytes and IS: (**A**) syringic acid; (**B**) ferulic acid; (**C**) caffeic acid; (**D**) vanillic acid; (**E**) *p*-coumaric acid; (**F**) 3,4-dihydroxybenzoic acid; (**G**) 4-hydroxy benzoic acid; (**H**) butylparaben.

To achieve better selectivity, the multiple reaction monitoring (MRM) mode with the precursor ions and product ions monitored for the seven compounds and the internal standard (IS), butylparaben were used. The analytes were tried in both positive and negative modes, and the negative ESI mode was selected for the higher response of signal intensity. Figure 4 shows the product ion scan spectra of the seven analytes and IS. The Supplementary Materials Table S1 displays the transitions and some relative parameters of these compounds.

### 2.2. Method Validation

#### 2.2.1. Selectivity

**Figure 2:**
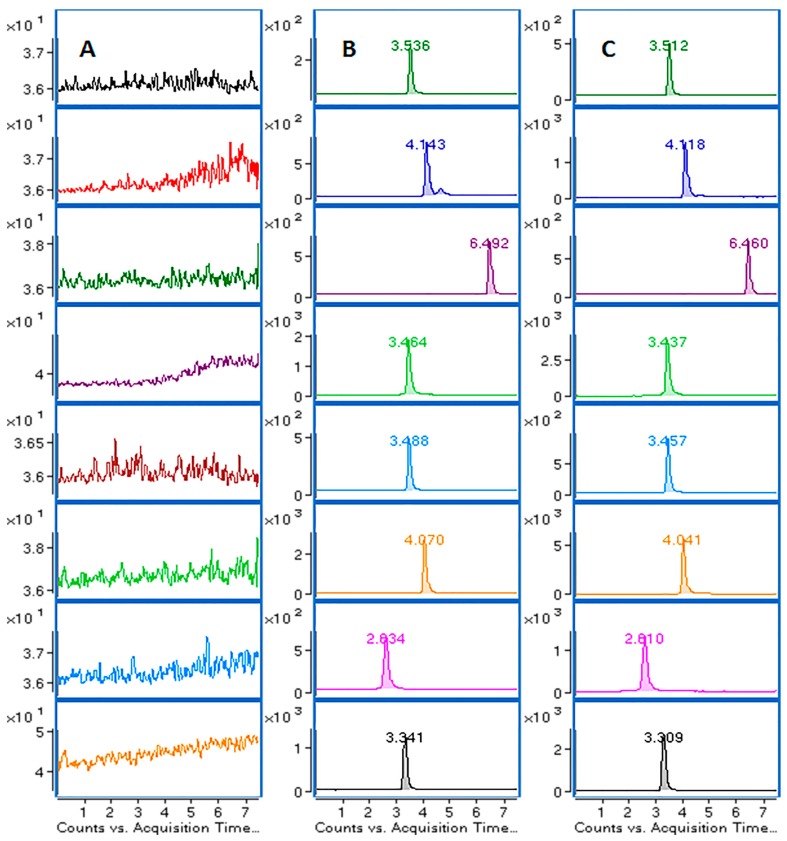
The chromatograms of the seven analytes and IS in rat plasma: (**A**) blank plasma; (**B**) blank plasma spiked with standard compounds (Medium Quality Control, MQC) and IS; (**C**) plasma sample from rat at 0.75 h after oral administration of *E. purpurea* extract; channel 1 for syringic acid; channel 2 for ferulic acid; channel 3 for IS; channel 4 for caffeic acid; channel 5 for vanillic acid; channel 6 for *p*-coumaric acid; channel 7 for 3,4-dihydroxybenzoic acid and channel 8 for 4-hydroxybenzoic acid.

The selectivity of the method was evaluated by analyzing blank rat plasma, blank plasma spiked with seven analytes and IS, and the plasma samples after oral administration of the *E. purpurea* extract in rats. The retention time of syringic acid, ferulic acid, caffeic acid, vanillic acid, *p*-coumaric acid, 3,4-dihydroxybenzoic acid, 4-hydroxybenzoic acid, and IS was 3.51 min, 4.12 min, 3.44 min, 3.46 min, 4.04 min, 2.61 min, 3.31 min and 6.46 min, respectively. Figure 2 shows the chromatographic profiles of blank plasma, blank plasma spiked with the analytes (Medium Quality Control, MQC) and IS, and rat plasma samples at 0.75 h after oral administration of *E. purpurea* extract. The results demonstrated that there was no significant interference from endogenous substances detected during the retention time of those phenolic acids.

#### 2.2.2. Linearity and Sensitivity

The calibration curves and linearity ranges for the seven phenolic acids are shown in the Supplementary Materials Table S2. All the analytes showed good linearity through concentration ranges with a correlation coefficient higher than 0.9945. The data demonstrated that the linear calibration ranges were 1.050~1050 ng/mL for syringic acid, 0.8320~832.0 ng/mL for ferulic acid, 0.8800~880.0 ng/mL for caffeic acid, 0.3264~326.4 ng/mL for vanillic acid, 0.8440~844.0 ng/mL for *p*-coumaric acid, 0.8080~808.0 ng/mL for 3,4-dihydroxybenzoic acid and 0.8560~856.0 ng/mL for 4-hydroxybenzoic acid. These results illustrated that the lower limit of quantification (LLOQ) of the seven compounds were all less than 1.050 ng/mL which was sufficient for pharmacokinetic studies of these phenolic acids in rat plasma.

#### 2.2.3. Accuracy and Precision

The intra-day and inter-day precision and accuracy of the samples, the spiked LLOQ and three quality control (QC) levels (Low Quality Control (LQC); Medium Quality Control (MQC); High Quality Control (HQC)), were measured for six replicates on the same day and also on three consecutive days. The results (Table 1) showed that the precision measurements were less than 14.74%, and accuracy ranged from −8.59% to 11.17% for the seven compounds, which illustrated that the method had considerable precision, accuracy and reproducibility.

#### 2.2.4. Extraction Recovery and Matrix Effect

The results of extraction recovery and the matrix effect (*n* = 6) are listed in Table 2. The mean extraction recoveries of the seven compounds at three concentrations of QC samples were more than 80%, and that of the IS was 86.55%. The observed matrix effects were between 81.17% and 102.9% within an acceptable range. These results indicated that there were no significant co-eluting substances influenced ionization of these analytes and IS.

#### 2.2.5. Stability

In terms of stability, the assay was applied to a variety of storage conditions. As is shown in Table 3, it was found that the QC samples at three concentrations in rat plasma were stable during three freeze-thaws and at room temperature for 4 h within the variation limits (RE% ≤ ±15%). Moreover, the long-term stability for two weeks at −20 °C was relatively stable. The data indicated that no significant degradation had been observed under these conditions.

### 2.3. Pharmacokinetic Studies

**Figure 3:**
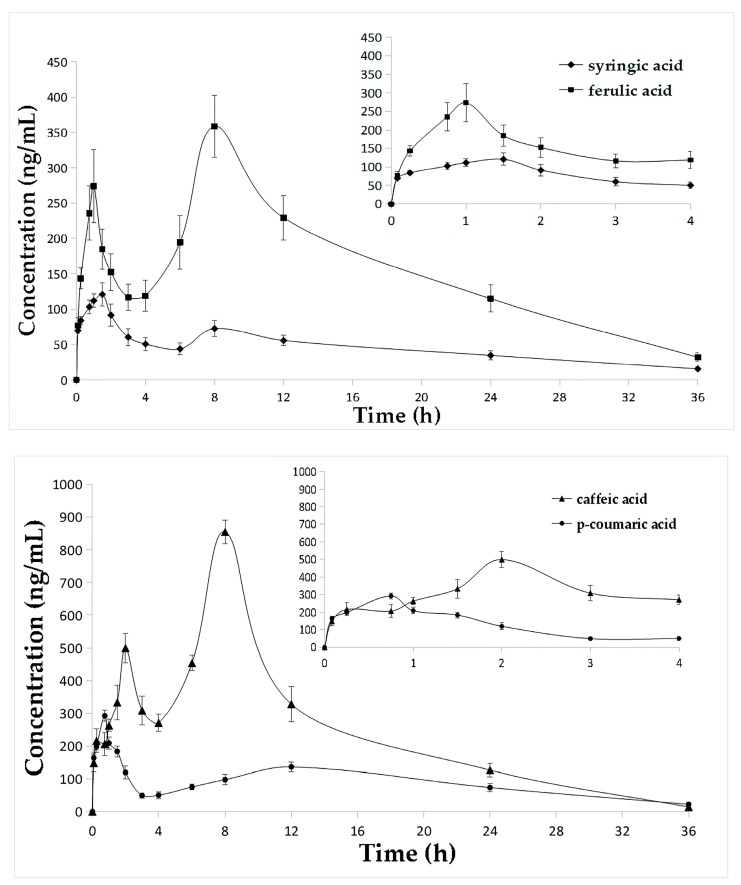

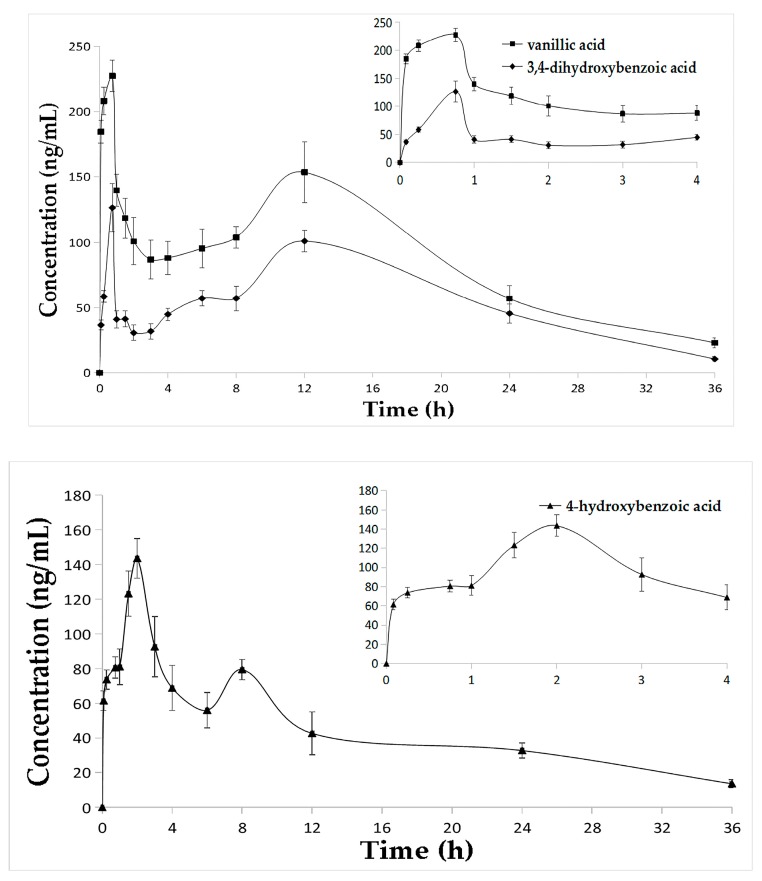
Mean concentration-time profiles of the seven analytes in rat plasma after oral administration of *E. purpurea* extract (*n* = 12, mean ± SD).

In this study, the validated UHPLC-ESI-MS/MS method was successfully applied in pharmacokinetic studies of the seven phenolic acids in 12 rats after oral administration of *E. purpurea* extract. Mean concentration-time profiles of the seven analytes in rat plasma are presented in Figure 3, and mean pharmacokinetic parameters are summarized in Table 4. The results showed that vanillic acid, *p*-coumaric acid and 3,4-dihydroxybenzoic acid were rapidly absorbed, the *T*_max_ for these compounds were 0.75 h, 0.75 h and 0.77 h, respectively. Meanwhile, the *T*_max_ of syringic acid and 4-hydroxybenzoic acid were 1.54 h and 1.96 h. The *T*_max_ of ferulic acid and caffeic acid were both 8 h, longer than the above compounds., and this was possibly due to the amount of the same parent components in the extract and hydrolysis of these two compounds at 8 h. While, *T*_1/2_ ranged from 5.31 h to 14.91 h, the *C*_max_ values varied in these analytes, 122.50 ± 16.43 ng/mL for syringic acid, 358.68 ± 43.84 ng/mL for ferulic acid, 854.61 ± 35.88 ng/mL for caffeic acid, 227.56 ± 11.94 ng/mL for vanillic acid, 292.51 ± 16.38 ng/mL for *p*-coumaric acid, 126.64 ± 18.07 ng/mL for 3,4-dihydroxybenzoic acid and 144.71 ± 11.62 ng/mL for 4-hydroxybenzoic acid. It was found that caffeic acid showed a different absorption from other compounds because of its high concentration in rat plasma. Moreover, AUC_0→t_ for these compounds ranged from 1643.96 ± 121.54 ng h/mL to 9217.13 ± 452.54 ng h/mL, and AUC_0→∞_ ranged from 1946.57 ± 164.14 ng h/mL to 9326.39 ± 441.68 ng h/mL. This may suggest that the absorption of caffeic acid was the highest among these analytes while syringic acid and 4-hydroxybenzoic acid were lowest. The results could help to investigate the action mechanism of these seven phenolic acids further and provided useful pharmacokinetic information about *E. purpurea*.

## 3. Discussion

### 3.1. The Content of Phenolic Acids in Echinacea purpurea

Although *E. purpurea* contain various components, phenolic acids are the main constituents in this plant. UHPLC-MS/MS was applied to determine the content of these phenolic acids in this study. We tested three batches of samples from Shanxi, Xinjiang and Hunan Province in China. And *E. purpurea* from Shanxi Province showed a higher content of these compounds than the other two after oral administration at the dose of 10 g/kg.

### 3.2. Selection of Extraction Method

Protein precipitation (PPT), solid phase extraction (SPE) and liquid–liquid extraction (LLE) are the most frequently used methods for sample preparations. PPT is evaluated for its convenient operation and high recovery rate, but it is not suitable for the low content of the analytes. Also, SPE easily prevents emulsification but it is time-consuming and results in expensive experimental costs. LLE was chosen for the preparation of plasma samples because of its lower potential for interfering compounds and higher sensitivity in this work. Ethyl acetate, ether, dichloromethane and acetone were used as an extraction solvent, the results found that ethyl acetate was an appropriate organic solvent with an acceptable extract efficiency and lower matrix effects for these analytes. In order to improve the extraction efficiency, 100 μL of 30% formic acid was added in plasma samples.

### 3.3. Selection of IS

It was important to choose an appropriate internal standard when performing the pharmacokinetic study. The selected internal standard should have analogical physicochemical properties and an approximate retention time. On one hand, the IS is not supposed to react with the analytes, on the other hand it should not interfere with the determination of target compounds. In this study, butylparaben was selected owing to the similarity of the analytes with an efficient ionization in the negative ionization mode. The results showed that butylparaben had no direct interference with analytes, thus it could be used as the IS to measure the concentration of these phenolic acids.

### 3.4. Pharmacokinetic Studies

It was found that all compounds had a double-peak phenomenon on the mean plasma concentration-time profiles in Figure 3, and this was probably due to the complexity of the gastrointestinal environment, that multiple absorption sites exist in different parts of the gastrointestinal tract, and their permeability of the drug is different [28,29]. However, it may be caused by enterohepatic circulation, the drug was secreted into the bile and a part of it was stored in the gallbladder, and when it contracted, it entered and was reabsorbed in the intestine again. In addition, the phenomenon of the double-peak of herbal medicine observed in pharmacokinetic study is more complicated. Most herbal medicines contain components that can be transformed into each other easily, causing some components to reappear.

## 4. Materials and Methods

### 4.1. Materials and Reagents

The reference compounds of ferulic acid and 3,4-dihydroxybenzoic acid were purchased from Ark Pharm (Chicago, IL, USA), caffeic acid was purchased from Aladdin Chemistry Company (Shanghai, China), and syringic acid, vanillic acid, 4-hydroxybenzoic acid and *p*-coumaric acid were supplied by Adamas Reagent Company (Basel, Switzerland). All standard substance purities were determined to be higher than 98%. Butylparaben was used as an internal standard (IS) which was provided by Guangfu Fine Chemical Research Institute (Tianjin, China). Methanol of HPLC-grade was obtained from Amethyst Chemicals (Beijing, China). HPLC-grade acetic acid was bought from ANPEL Scientific Instrument Company (Shanghai, China). Ultra-pure water for the UHPLC mobile phase was prepared by a Milli-Qwater purification system (Millipore, Molsheim, France).

*E. purpurea* was purchased from Xi’an Ruibo Biological Technology Co. Ltd. (Xi’an, China) in October, 2016 and identified by Prof. Li Yao in Harbin Medical University.

### 4.2. Preparation of E. purpurea

For preparing the *E. purpurea* extract, 500 g of the medicine materials were extracted under reflux with 5 L ethanol-water (60:40, *v*/*v*) at 50 °C, three times, for 2 h each time. Then the extract was combined, filtered and condensed until it became dry. The residue was dissolved by proper amounts of water and the prepared *E. purpurea* extract of 0.5 g/mL was stored in a refrigerator at 4 °C when it was not used. The contents of the seven phenolic acids were quantitatively determined by UHPLC-MS/MS; syringic acid was 0.109 mg/mL, ferulic acid was 0.365 mg/mL, caffeic acid was 1.908 mg/mL, vanillic acid was 0.228 mg/mL, *p*-coumaric acid was 0.089 mg/mL, 3,4-dihydroxybenzoic acid was 0.342 mg/mL, and 4-hydroxybenzoic acid was 0.136 mg/mL.

### 4.3. Preparation of Standard Solutions and Quality Control Samples

All solutions and IS were prepared in methanol. A mixed stock solution containing 10,500 ng/mL of syringic acid, 8320 ng/mL of ferulic acid, 8800 ng/mL of caffeic acid, 3264 ng/mL of vanillic acid, 8440 ng/mL of *p*-coumaric acid, 8080 ng/mL of 3,4-dihydroxybenzoic acid, and 8560 ng/mL of 4-hydroxybenzoic acid were prepared. Stock solutions of IS were obtained and diluted to a 2120 ng/mL working solution which was stored at 4 °C. The calibration samples were prepared by spiking a series of mixture standard solution into 100 μL blank rat plasma at concentrations of 1.050, 5.250, 13.13, 32.81, 65.63, 131.3, 262.5 and 1050 ng/mL for syringic acid; 0.832, 4.160, 10.40, 26.00, 52.00, 104.0, 208.0 and 832.0 ng/mL for ferulic acid; 0.880, 4.400, 11.00, 27.50, 55.00, 110.0, 220.0 and 880.0 ng/mL for caffeic acid; 0.3264, 1.632, 4.080, 10.20, 20.40, 40.80, 81.60 and 326.4 ng/mL for vanillic acid; 0.844, 4.220, 10.55, 26.38, 52.75, 105.5, 211.0 and 844.0 ng/mL for *p*-coumaric acid; 0.808, 4.040, 10.10, 25.25, 50.50, 101.0, 202.0 and 808.0 ng/mL for 3,4-dihydroxybenzoic acid; 0.856, 4.280, 10.70, 26.75, 53.50, 107.0, 214.0 and 856.0 ng/mL for 4-hydroxybenzoic acid. Quality control (QC) samples were prepared in blank plasma at four different concentrations levels for each analytes, high QC (840.0, 665.6, 704.0, 261.1, 675.2, 646.6 and 684.8 ng/mL), medium QC (65.63, 52.00, 55.00, 20.40, 52.75, 50.50 and 53.50 ng/mL), low QC (5.250, 4.160, 4.400, 1.632, 4.220, 4.040 and 4.280 ng/mL) and lower limits of quantification (LLOQ) (1.050, 0.8320, 0.8800, 0.3264, 0.8440, 0.8080 and 0.8560 ng/mL), for syringic acid, ferulic acid, caffeic acid, vanillic acid, *p*-coumaric acid, 3,4-dihydroxybenzoic acid and 4-hydroxybenzoic acid, respectively. All stock working solutions were kept at 4 °C before they were used.

### 4.4. Animal Experiments and Plasma Sample Extraction Procedure

Twelve male Sprague-Dawley (SD) rats (body weight 220 ± 20 g) were provided by Heilongjiang University of Chinese Medicine (Harbin, China). Each rat fasted 12 h before administration, with free drinking during the experiment. Blood samples were obtained from rats after oral administration of the extract of *E. purpurea* with a dose of 10 g/kg body weight [30]. Blood samples (0.25 mL) of the retinal venous plexus were taken at 0.083, 0.25, 0.75, 1.0, 1.5, 2, 3, 4, 6, 8, 12, 24 and 36 h, then moved immediately into a 0.5 mL centrifuge tube that was spread with heparin at each time point. Meanwhile, the animal blood was centrifuged at 13,000 rpm for 5 min at 4 °C. The plasma was frozen in a −20 °C refrigerator in case of deterioration.

First, the frozen plasma was thawed at room temperature and then a liquid–liquid extraction (LLE) method was utilized. Then, a 100 μL aliquot of each plasma sample was removed except for the calibration standards which was mixed with 10 μL IS into a 10 mL glass tube. We continued by adding 100 μL methanol and 100 μL 30% formic acid, vortex-mixing for 1 min, then extracted 3 mL ethyl acetate, vortex-mixing for 3 min, centrifuged for 5 min (3500 rpm), and transferred the upper organic layer into a new tube. The layer of ethyl acetate was dried with a stream of nitrogen, the dry residue was dissolved with a 100 μL mobile phase, vortexed for 2 min and samples were filtered by a 0.22 μm microporous membrane. A 10 μL aliquot was injected into the UHPLC-ESI-MS/MS system [27].

### 4.5. UHPLC-ESI-MS/MS Conditions

The UHPLC system consisted of an Agilent series 1290 system (Agilent, Santa Clara, CA, USA) equipped with (Agilent, Santa Clara, CA, USA) an auto-sample, a quaternary pump, and a column oven. During the analysis, the column was kept at 30 °C and the flow rate was 0.3 mL/min. The mobile phase used 0.1% acetic acid in water (A) and methanol (B) as follows: 0–4 min, 20–75% (B); 4–5.5 min, 75–80% (B), 5.5–6.0 min, 80–90% (B); 6–7 min 90% (B). The injection volume of samples was 10 μL. The outlet column pressure was 480 bar under these chromatographic conditions, and the pressure limit of the system was 1200 bar.

The UHPLC system was coupled to a 6430 triple-quadrupole mass spectrometer with an ESI interface (Agilent, Santa Clara, CA, USA). Ionization was done by electrospray ionization in the negative mode. Seven compounds and IS were collected in the multiple reaction monitoring (MRM) mode. The optimized MS/MS parameters were as follows: spray voltage, 3500 V; gas temperature, 355 °C with a flow rate of 400 L/h; nitrogen (N_2_) used as cone gas with a flow rate of 11 L/h; and N_2_ (99.99% purity) used as the nebulizer gas set at 30 psi. The transitions of protonated charged molecular ions were *m*/*z* 197.1→181.9 for syringic acid, *m*/*z* 193.1→133.9 for ferulic acid, *m*/*z* 179.1→134.9 for caffeic acid, *m*/*z* 167.1→152.0 for vanillic acid, *m*/*z* 163.1→118.9 for *p*-coumaric acid, *m*/*z* 153.0→108.9 for 3,4-dihydroxybenzoic acid, *m*/*z* 137.0→93.0 for 4-hydroxybenzoic acid and *m*/*z* 193.0→91.9 for IS, respectively. Product ion mass spectra of seven analytes and IS are exhibited in Figure 4. Other MRM parameters are displayed in Table S1.

### 4.6. Method Validations

The assay was validated by measuring its selectivity, linearity, precision, accuracy, extraction recovery, matrix effect and stability in accordance to the FDA guidelines, http://www.fda.gov/downloads/Drugs/GuidanceComplianceRegulatoryInformation/Guidances/UCM368107.pdf [31].

#### 4.6.1. Selectivity

Testing for selectivity was carried out by comparing the chromatograms of twelve blank plasma samples, blank plasma samples spiked with seven analytes with IS and plasma samples obtained from rats after oral administration of *E. purpurea* extract. For all blank plasma samples it was ascertained whether endogenous constituents and other substances existing in the samples would interfere in the detection of the analytes and IS.

#### 4.6.2. Linearity and Sensitivity

The calibration curves were constructed by plotting the peak area ratios of analytes and IS (*y*) versus each nominal concentration (*x*) of the analytes. The calibration model was selected based on the analysis of the data by linear regression with weighting factors (1/*x*^2^). The LLOQ was the lowest concentration of analytes in a sample where the acceptable precision expressed as relative standard deviation (RSD) ranged from 80% to 120%, and the accuracy expressed as relative error (RE) was within ±20%. The signal-to-noise (S/N) ratio should be greater than 10 for a quantitative assay.

#### 4.6.3. Accuracy and Precision

The accuracy and precision of intra-day and inter-day were calculated by analyzing six replicates of LLOQ and QC samples at three concentrations (LQC, MQC, HQC) on the same day and three batches on three consecutive days. The precision was indicated as the relative standard deviation (RSD) which should not be more than 15%. The data accuracy was expressed as the percentage of relative error (RE) which should also be within ±15%.

#### 4.6.4. Extraction Recovery and Matrix Effect

The extraction recovery and matrix effect assays at three concentrations were determined in sets of six replicates in the same run. To assess the extraction recovery, seven analytes were determined by comparing the peak areas of an extracted sample against a post-extraction spiked sample and calculated by the ratio of the peak responses. The matrix effect was evaluated by comparing the absolute peak areas of blank matrix samples spiked after extraction with analytes to pure solution of the analytes.

#### 4.6.5. Stability

The stability of the seven analytes in rat plasma was evaluated under a variety of storage conditions with three concentrations of QC samples of six replicates under the following conditions: the freeze-thaw stability was investigated after three cycles of being stored at −20 °C then thawed at ambient temperature, the room temperature stability was tested by storage for 4 h at room temperature, and long-term stability was also tested by assaying frozen QC samples after storage at −20 °C for 2 weeks. The post-preparation samples were analyzed after storage for 12 h at 4 °C. The peak area ratio response of stability samples was determined and versus those freshly prepared. For all stability results, the analyzing samples were considered stable when the percentage of relative error (RE) was within ±15% compared with freshly prepared QC samples.

### 4.7. Pharmacokinetic Studies

The rat plasma concentration-time curves of seven compounds were plotted. After oral administration of *E. purpurea* extract in twelve rats, the maximum drug concentration in rat plasma (*C*_max_) and the time to reach maximum drug concentration (*T*_max_) were directly obtained from concentration-time data. The elimination rate constant (*Ke*) was calculated by linear regression of the terminal points in a semi-log plot of the plasma concentration against time. The elimination half-life (*T*_1/2_) was calculated by the formula *T*_1/2_ = 0.693/*Ke*. Other pharmacokinetic parameters including the area under the concentration-time curve (AUC_0→t_), and the area under the plasma concentration-time curve to time infinity (AUC_0→∞_) were calculated using non-compartmental pharmacokinetics analysis.

## 5. Conclusions

For the first time, an UHPLC-ESI-MS/MS method was established and validated to simultaneously determine seven phenolic acids (syringic acid, ferulic acid, caffeic acid, vanillic acid, *p*-coumaric acid, 3,4-dihydroxybenzoic acid and 4-hydroxybenzoic acid) in rat plasma. In addition, the method was rapid, sensitive, specific, accurate and reproducible for measurements of these compounds. Pharmacokinetic results were successfully obtained after oral administration of *E. purpurea* extract in rats. The study will be particularly helpful when applied to preclinical pharmacokinetic studies in rats and for collecting useful information for guiding clinical applications of *E. purpurea*.

## Figures and Tables

**Table 1:** The intra-day and inter-day precision and accuracy of the seven analytes.

Compounds	Spiked Conc (ng/mL)	Measured Conc (ng/mL)	Accuracy (%)	Intra-Day Precision (%)	Inter-Day Precision (%)
Syringic acid	1.050	1.08 ± 0.13	2.66	6.28	12.61
	5.250	5.14 ± 0.34	−2.12	6.99	3.89
	65.63	64.14 ± 4.30	−1.39	7.05	1.37
	840.0	802.1 ± 44.09	−4.51	5.64	4.28
Ferulic acid	0.832	0.85 ± 0.10	2.52	12.60	8.23
	4.160	4.00 ± 0.43	−3.80	11.03	8.98
	52.00	53.41 ± 3.47	2.71	4.82	13.60
	665.6	608.4 ± 36.12	−8.59	4.42	12.39
Caffeic acid	0.880	0.86 ± 0.09	−2.04	9.98	13.93
	4.400	4.71 ± 0.31	7.09	7.03	2.16
	55.00	55.55 ± 3.74	1.01	7.10	2.84
	704.0	701.2 ± 29.50	−0.39	4.26	3.75
Vanillic acid	0.326	0.36 ± 0.03	10.93	9.04	13.26
	1.632	1.62 ± 0.16	−0.59	9.32	13.00
	20.40	20.72 ± 1.77	1.57	8.53	8.72
	261.1	255.9 ± 14.17	−1.45	4.82	9.30
*p*-Coumaric acid	0.844	0.94 ± 0.10	11.17	11.10	10.48
	4.220	3.97 ± 0.39	−5.97	10.22	5.47
	52.75	51.93 ± 5.04	−1.55	9.81	8.81
	675.2	632.7 ± 50.98	−6.30	8.33	5.65
3,4-Dihydroxy benzoic acid	0.808	0.78 ± 0.06	−3.27	7.11	10.18
	4.040	4.03 ± 0.42	−0.33	10.77	6.11
	50.50	53.82 ± 5.71	6.57	10.71	9.81
	646.4	663.5 ± 45.89	2.46	6.95	6.67
4-Hydroxy benzoic acid	0.856	0.84 ± 0.12	−1.52	14.74	9.14
	4.280	4.25 ± 0.47	−0.76	11.41	7.89
	53.50	53.60 ± 5.80	0.18	11.35	5.38
	684.8	683.2 ± 66.79	−0.23	10.11	6.71

**Table 2:** Matrix effect and extraction recovery for the seven analytes and IS (*n* = 6).

Compounds	Spiked Conc (ng/mL)	Matrix Effect		Extraction Recovery	
		Mean (%)	RSD (%)	Mean (%)	RSD (%)
Syringic acid	5.250	86.06	14.39	89.96	6.18
	65.63	84.84	5.71	92.25	10.45
	840.0	81.17	12.72	90.45	4.72
Ferulic acid	4.160	82.87	13.68	94.50	7.65
	52.00	91.19	12.57	87.47	4.02
	665.6	100.2	10.92	95.79	13.19
Caffeic acid	4.400	94.75	9.79	81.43	12.43
	55.00	97.88	11.04	95.03	11.56
	704.0	102.9	12.46	96.17	11.26
Vanillic acid	1.632	89.37	12.80	82.27	12.52
	20.40	100.0	11.00	89.38	11.82
	261.1	83.94	14.43	95.85	11.83
*p*-Coumaric acid	4.220	101.7	10.93	98.08	14.44
	52.75	101.3	11.40	96.40	12.72
	675.2	97.20	11.21	94.63	11.68
3,4-Dihydroxybenzoic acid	4.040	99.45	14.28	87.88	12.85
	50.50	96.16	9.03	84.62	14.87
	646.4	88.73	10.49	89.98	11.82
4-Hydroxybenzoic acid	4.280	102.4	12.80	80.28	10.51
	53.50	88.88	11.00	85.06	6.74
	684.8	93.22	9.72	90.83	6.25
IS	212.0	95.74	8.95	86.55	7.63

**Table 3:** The stability of the seven analytes under different storage conditions (*n* = 6).

Compounds	Spiked Conc (ng/mL)	Stability (RE%)			
		Freeze-Thaw	Short-Term	Long-Term	Post-Preparative
Syringic acid	5.250	−3.75	−1.95	−0.66	−10.74
	65.63	4.48	−1.50	−0.79	−7.64
	840.0	−2.61	−5.73	−1.30	−6.08
Ferulic acid	4.160	−7.10	−4.21	7.93	−0.89
	52.00	−3.80	4.61	6.80	4.84
	665.6	−13.12	−8.77	−1.89	−4.91
Caffeic acid	4.400	7.79	7.45	6.01	10.35
	55.00	2.18	−0.16	1.00	3.89
	704.0	6.15	6.12	−1.54	9.83
Vanillic acid	1.632	−6.56	2.24	2.91	9.11
	20.40	5.74	−0.67	−0.37	−6.34
	261.1	5.28	1.59	−0.32	−8.03
*p*-Coumaric acid	4.220	−7.57	−3.59	−6.75	8.52
	52.75	−0.82	1.57	−5.40	11.50
	675.2	−8.78	−4.81	−5.30	7.12
3,4-Dihydroxy benzoic acid	4.040	−0.11	2.03	−2.93	9.29
	50.50	6.57	10.18	2.30	−5.47
	646.4	−0.52	4.80	3.65	−4.01
4-Hydroxybenzoic acid	4.280	10.03	10.62	−3.31	−6.42
	53.50	2.63	−0.46	0.21	10.47
	684.8	−1.01	−2.50	2.81	7.71

**Table 4:** Pharmacokinetic parameters of the seven compounds in rat plasma after oral administration of *E. purpurea* extract (*n* = 12, mean ± SD).

Compounds	*C*_max_ (ng/mL)	*T*_max_ (h)	*T*_1/2_ (h)	AUC_0→t_ (ng h/mL)	AUC_0→∞_ (ng h/mL)
Syringic acid	122.50 ± 16.43	1.54 ± 0.14	13.21 ± 1.66	1645.22 ± 132.79	1946.57 ± 164.14
Ferulic acid	358.68 ± 43.84	8.00 ± 0	8.48 ± 0.71	5620.34 ± 453.15	6015.42 ± 497.85
Caffeic acid	854.61 ± 35.88	8.00 ± 0	5.31 ± 0.50	9217.13 ± 452.54	9326.39 ± 441.68
Vanillic acid	227.56 ± 11.94	0.75 ± 0	8.89 ± 1.12	3136.01 ± 263.85	3435.67 ± 233.61
*p*-Coumaric acid	292.51 ± 16.38	0.75 ± 0	9.32 ± 1.29	3132.51 ± 156.23	3439.38 ± 217.96
3,4-Dihydroxy benzoic acid	126.64 ± 18.07	0.77 ± 0.07	7.45 ± 0.87	1934.80 ± 123.40	2051.96 ± 115.85
4-Hydroxy benzoic acid	144.71 ± 11.62	1.96 ± 0.14	14.91 ± 5.37	1643.96 ± 121.54	1947.84 ± 175.74

## Supplementary Materials

Supplementary Materials are available online, Table S1: Multiple reaction monitoring (MRM) parameters of the seven analytes and IS, Table S2: The calibration curves, linear ranges and lower limits of quantification (LLOQ) of the seven analytes.
