# Supplementary material for: Simultaneous Determination of Seven Phenolic Acids in Rat Plasma Using UHPLC-ESI-MS/MS after Oral Administration of Echinacea purpurea Extract
Source: Molecules. 2017 Sep 7;22(9):1494. doi: 10.3390/molecules22091494 (PMC6151385; doi:10.3390/molecules22091494)
Supplement: Supplementary file 1 [file molecules-22-01494-s001.pdf]

Table S1. Multiple reaction monitoring (MRM) parameters of the seven analytes and IS.

Compounds	Ion pair	Fragmentor (V)	Collision energy (V)	Cell accelerator voltage	Polarity
Syringic acid	197.1→181.9	100	7	3	Negative
Ferulic acid	193.1→133.9	100	12	3	Negative
Caffeic acid	179.1→134.9	112	12	3	Negative
Vanillic acid	167.1→152.0	85	10	3	Negative
p-Coumaric acid	163.1→118.9	95	12	3	Negative
3,4-Dihydroxybenzoic	153.0→108.9	93	10	3	Negative
4-Hydroxybenzoic acid	137.0→93.0	75	12	3	Negative
IS	193.0→91.9	110	20	3	Negative

Table S2. The calibration curves, linear ranges and lower limits of quantification (LLOQs) of the seven analytes.

Compounds	Regression Equation	R^2	Linear Ranges (ng/mL)	LLOQ (ng/mL)
Syringic acid	Y=0.0002X+0.0007	0.9988	1.050~1050	1.050
Ferulic acid	Y=0.0010X+0.0081	0.9997	0.8320~832.0	0.8320
Caffeic acid	Y=0.0011X+0.0018	0.9957	0.8800~880.0	0.8800
Vanillic acid	Y=0.0005X+0.0054	0.9945	0.3264~326.4	0.3264
p-Coumaric acid	Y=0.0022X+0.0310	0.9972	0.8440~844.0	0.8440
3,4-Dihydroxybenzoic acid	Y=0.0010X+0.0033	0.9982	0.8080~808.0	0.8080
4-Hydroxybenzoic acid	Y=0.0013X+0.0252	0.9979	0.8560~856.0	0.8560
